# Towards resolving the phosphorus chaos created by food systems

**DOI:** 10.1007/s13280-019-01255-1

**Published:** 2019-09-21

**Authors:** Paul J. A. Withers, Kirsty G. Forber, Christopher Lyon, Shane Rothwell, Donnacha G. Doody, Helen P. Jarvie, Julia Martin-Ortega, Brent Jacobs, Dana Cordell, Myles Patton, Miller A. Camargo-Valero, Rachel Cassidy

**Affiliations:** 1 Lancaster Environment Centre, Lancaster University, Lancaster, LA1 4YQ UK; 2 Sustainability Research Institute, University of Leeds, Leeds, LS2 9TJ UK; 3 Agri-Food and Bioscience Institute, Belfast, BT9 5BX UK; 4 Centre for Ecology and Hydrology, Wallingford, Oxfordshire OX10 8BB UK; 5 Institute for Sustainable Futures, University of Technology, Sydney, Australia; 6 BioResource Systems Research Group, School of Civil Engineering, University of Leeds, Leeds, LS2 9JT UK

**Keywords:** Efficiency, Eutrophication, Food system, Phosphorus, Stakeholders, Sustainability

## Abstract

The chaotic distribution and dispersal of phosphorus (P) used in food systems (defined here as disorderly disruptions to the P cycle) is harming our environment beyond acceptable limits. An analysis of P stores and flows across Europe in 2005 showed that high fertiliser P inputs relative to productive outputs was driving low system P efficiency (38 % overall). Regional P imbalance (P surplus) and system P losses were highly correlated to total system P inputs and animal densities, causing unnecessary P accumulation in soils and rivers. Reducing regional P surpluses to zero increased system P efficiency (+ 16 %) and decreased total P losses by 35 %, but required a reduction in system P inputs of ca. 40 %, largely as fertiliser. We discuss transdisciplinary and transformative solutions that tackle the P chaos by collective stakeholder actions across the entire food value chain. Lowering system P demand and better regional governance of P resources appear necessary for more efficient and sustainable food systems.

**Electronic supplementary material:**

The online version of this article (10.1007/s13280-019-01255-1) contains supplementary material, which is available to authorized users.

## Introduction

Enrichment of the hydrosphere with phosphorus (P) used in global food production is compromising water quality and biodiversity and the provision of linked ecosystem services (MacDonald et al. 2016; Campbell et al. 2017). Mitigating the negative ecosystem impacts and high societal costs of this eutrophication requires reductions in P loading to inland and coastal waterbodies (Schindler et al. 2016), but achieving these reductions in the face of continuing population growth, increasing food demand and future climate change is however problematic (Ockenden et al. 2017; Springmann et al. 2018). Whilst some rivers and lakes in developed regions have seen welcome declines in their P status as a result of reductions in sewage effluent discharges (e.g. European Environment Agency 2015), rates of annual P storage in catchments remain high because riverine P export is considerably less than the net anthropogenic P inputs entering catchments (Powers et al. 2016).

This continued and highly variable accumulation of P in catchments occurs over multiple timescales and represents a long-term ‘legacy’ source of P losses to waterbodies which is difficult to mitigate (Jarvie et al. 2013; Abbott et al. 2018). Losses of P in land runoff occur from multiple point and diffuse sources across urban and rural landscapes, in a spectrum of particulate and soluble P forms (Withers and Bowes 2018). Anthropogenic P inputs have consequently become widely and chaotically dispersed across both terrestrial and aquatic environments. In this context, chaos can be defined as the disorderly disruption of natural P cycling that increases the biosphere’s sensitivity to small changes in environmental conditions. The chaotic dispersal of P in landscapes and waterscapes, in turn, reflects the chaotic governance of P in terms of its distribution, usage, loss and accumulation within society, the majority of which occurs within the food system (e.g. van Dijk et al. 2016). Geographical segregation of crop and livestock production systems, increasing urbanisation and global trade in food commodities have totally disrupted local production and P cycles in unforeseen ways driven by market forces (e.g. Jarvie et al. 2015; Nesme et al. 2018). This disorderly anthropogenic enrichment of our environment threatens further transition of socio-ecological systems into chaotic states that are very difficult to predict; for example in sudden ecosystem shifts to less desired states (Folke et al. 2004), or in increased vulnerability to a future scarcity of finite phosphate rock reserves (Cordell and Neset 2014).

Attempts to improve P-use efficiency and sustainability within food systems and limit P losses to water have hitherto typically been reductionist in character. They have focused on improved wastewater treatment to remove P in effluent discharge (Schindler et al. 2016), and altering farmland and landscape management to avoid overuse of P inputs on farms and reduce the mobilisation and delivery of P in runoff from agricultural land (Kleinman et al. 2015). A focus on field and farm-scale P management is understandable because modern farming practices have increased land vulnerability to P loss in runoff and erosion (Withers and Bowes 2018), and that is the scale at which P is currently managed through farmer choice and decisions. However, sole reliance on agronomic solutions to P source management ignores the P inefficiencies, wastage and losses that occur at other stages in the food supply chain; for example, during the mining of phosphate rock, in food processing and arising from our consumption of food (Cordell and White 2014; Jurgilevich et al. 2016). These wider food chain inefficiencies are a consequence of societal functioning involving a wider range of stakeholders, and are therefore largely outside the control of the producer.

In this paper, we examine the distribution and dispersal of P used in Europe’s food systems and consider some causal drivers of poor environmental performance. We find that system P inefficiencies and losses are related to the P-input pressure imposed by the organisation of the food system, and argue for a more transdisciplinary, transformative and system-wide approach to P governance and management that addresses the regional P imbalance that is the root cause of P unsustainability (Abson et al. 2016; Gordon et al. 2017). We consider the need for a societal response to this complex and dynamic socio-environmental issue based on the transformational potential of all stakeholders in the food chain, and discuss potential leverage mechanisms for transformative change towards improved resource conservation, environmental performance and long-term sustainability.

## Methods

Efficiencies of P use, surplus soil P accumulation and P losses from the food system were compared for the EU 27 countries based on the national P flows dataset for 2005 compiled using substance flow analysis (SFA) by van Dijk et al. (2016). This seminal SFA provides national data on annual P inputs, P outputs and internal P cycling by total amount (Gg), and on an areal (kg P ha^−1^) and a per capita basis (kg P ca^−1^), Fig. S1. System P efficiency assessed productive P output as a function of P inputs and was defined as human consumption of P + P exports divided by the P imports into the food system (fertiliser, feed and food). Whole system P efficiency was further sub-divided into sector efficiencies (calculated as consumed or exported P divided by imported and recycled P) for crop production, animal production and food processing. System surplus P was defined as the annual amount of unused P that accumulated in the soil each year as detailed in van Dijk et al. (2016). The P surplus represents the imbalance or difference between total P inputs (i.e. imports) and total P outputs (i.e. P exports and P losses), Fig. S1. Total losses of P from the food system included wastewater discharges from households and industry, waste disposal via incineration and landfill and diffuse P losses in land runoff, all based on measured national data for each sector (households, food processing, livestock and crop production, Fig. S1) as reviewed by van Dijk et al. (2016). Specific sub-losses of P to water which included direct municipal wastewater discharges (termed effluent losses), runoff losses from livestock hardstandings and runoff, leaching and soil erosion from agricultural soils (termed soil losses) were also calculated. Flows of P into and out of the non-food system in each country were excluded from the calculations. In addition to land area and population, national data on arable land (including temporary grass), permanent pasture, total livestock units, and wealth (taken as Gross Domestic Product) for 2005 were obtained from the Eurostat pocketbook database (European Commission 2007) to help identify some causal relationships and efficiency indicators.

Efficiency, surplus and P loss metrics by area and per capita were compared to national indicators by single and multiple linear regression analysis using SPSS v25. Malta was excluded from areal comparisons due to their exceptionally small area relative to their P flows. Non-linear fitting was preferred where this gave a significant (*P* < 0.05) improvement over a linear function in the variance accounted for. To aid interpretation, the EU 27 countries were allocated to Western, Eastern, Northern and Southern regions of Europe according to their climate zones (Table S1).

The dispersal and accumulation of food system P inputs into terrestrial and aquatic landscapes was assessed by the variability in bioavailable P in EU soils and rivers. Dispersal of P in soils was quantified by the variation in the mean concentrations of Olsen-extractable P in cropland and grassland sampled across Europe in 2009 (and part 2012) as part of the LUCAS survey (Tóth et al. 2013). Data were not available for Bulgaria, Cyprus, Luxembourg, Malta and Romania. Olsen-P is a metric of crop-available inorganic P reserves in soil arising from the cumulative P surpluses generated over time in farming systems (Tóth et al. 2013). This metric is not always the best predictor of crop P availability, depending on soil type, but nevertheless provides a common measure of soil P fertility status across Europe. A mean agronomic optimal Olsen-P for Europe of 20 mg kg^−1^ (Nawara et al. 2017) was used to illustrate how much Olsen-P has deviated from the level required for optimal growth of grass and arable crops. Full descriptive statistics of all national soil survey data used in this analysis are given in Table S2.

Dispersal of P in EU rivers was quantified by the mean concentrations of soluble reactive P (SRP) relative to nitrate-N (NO_3_N) measured as part of the Waterbase survey v 14 (European Environment Agency 2018b). The values represent the mean of all national river SRP and NO_3_N data submitted to the European Commission for the period 2003–2007 (or closest to this). This five-year period was chosen as it spans the year the national food system flows were calculated by van Dijk et al. (2016). No data were available for Malta. As these data are produced using different methodologies, additional data are also given on mean SRP and NO_3_–N concentrations in different river and land runoff typologies representative of lowland England using standardised catchment monitoring protocols (Withers et al. 2009; Neal et al. 2012). A mean eutrophication control target of 0.06 mg SRP L^−1^ was used here to illustrate the extent of impairment of freshwaters with respect to readily bioavailable P (Withers and Bowes 2018). Full descriptive statistics of all national river and runoff survey data used in this analysis are given in Tables S3–S5.

## Results

**Fig. 1:**
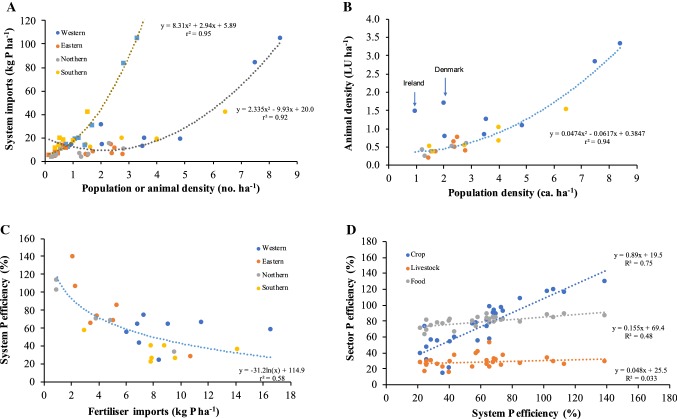
Imports of P into the food system defining the P-input pressure which is a function of population density and animal density (**a**). Denmark and Ireland have higher animal densities relative to the human population (**b**) because of the dominance of the livestock sector in their agricultural systems. System P efficiency is largely controlled by fertiliser imports (**c**), because of the dominant influence of cropland efficiency on overall system efficiency (**d**). For definitions of system and sector efficiencies, see the text. Data are derived from the P flows in 2005 across the EU 27 countries compiled by van Dijk et al. (2016) and EU demographical data (European Commission 2007) for 2005

Total annual imports of P into the food system (here also termed P-input pressure) ranged up to 144 kg P ha^−1^ and 15 kg P ca^−1^ across the EU 27. Fertiliser was often the largest P import (64 % for all Europe), but imported animal feed was the largest P import in the Czech Republic, Denmark and Slovenia, whilst food P imports dominated in Belgium, Cyprus, Estonia, Latvia, Luxembourg, Malta and The Netherlands. Multiple regression analysis showed that total system P imports were primarily dependent on animal densities (*P* < 0.001), which were closely linked to population densities (Fig. 1A, B). At the national level, fertiliser P imports were also related to animal densities (*P* < 0.001, *r*^2^ = 0.43) rather than to agricultural land area.

Whole system P efficiency varied from 22 to 139 % within individual countries, and was 38 % for all Europe. Values above 100 % (i.e. P outputs > P inputs) occurred more in North-Eastern Europe where large reductions in cropland area and fertiliser use have occurred since the fall of communism (Fig. 1C; Kuemmerle et al. 2017). However, P imports exceeded productive P outputs in the majority of EU countries (Table 1). Imported fertiliser P was the best predictor of whole system P efficiency (Table 2 and Fig. 1C), with lowest P efficiencies occurring in Spain (22 %), Ireland (24 %), Portugal (26 %), Greece (26 %) and Poland (29 %) because agricultural P output per hectare was low despite high fertiliser P inputs.

**Table 1:** Food system’s general characteristics, P flows and efficiency metrics across the EU in 2005. Data are derived from the P flows in 2005 across the EU 27 countries compiled by van Dijk et al. (2016) and EU data on crop area and animal numbers (European Commission 2007) for 2005. For definitions of system inputs, productive outputs, system losses, efficiency and surplus metrics, see text and Fig. S1. *M* million, *LU* Livestock units, *Gg* Gigograms

Country	Characteristics				P flows			Metrics				Surplus (kg P ha^−1^)
	Land	Animals	People	GDP	Inputs	Outputs	Losses	Efficiency ( %)				
	(M ha)	(M LU)	(M)	(€ × 10^12^)	(Gg)	(Gg)	(Gg)	System	Crop	Animal	Food	
Belgium	1.39	3.88	10.41	0.335	115.4	67.5	28.9	59	54	41	84	23.25
Bulgaria	5.26	0.87	7.74	0.029	26.5	19.4	16.6	73	96	24	81	− 0.07
Czech Republic	4.26	2.06	10.25	0.127	25.7	27.3	21.4	106	118	28	84	− 2.11
Denmark	2.69	4.52	5.42	0.227	81.3	35.4	26.9	44	58	36	66	10.10
Germany	16.99	18.12	82.59	2.423	315.5	235.2	158.9	75	91	36	81	1.77
Estonia	0.85	0.31	1.35	0.015	4.3	4.4	2.9	102	116	32	87	− 1.10
Ireland	4.29	6.20	4.16	0.191	57.2	14.0	19.6	24	73	16	62	6.73
Greece	6.86	2.46	11.18	0.228	77.8	20.1	36.2	26	46	24	68	5.92
Spain	28.95	14.40	43.38	1.051	387.3	85.1	115.2	22	38	28	71	9.00
France	29.56	22.66	60.98	1.892	406.1	260.6	131.6	64	80	25	82	3.77
Italy	14.61	9.54	58.76	1.536	263.2	106.4	125.9	40	53	28	81	7.49
Cyprus	0.16	0.24	1.03	0.016	6.7	2.4	2.3	36	14	22	76	28.91
Latvia	1.75	0.41	2.30	0.021	5.3	6.0	4.2	113	115	25	88	− 0.91
Lithuania	2.75	1.12	3.41	0.028	16.4	11.5	7.1	70	88	30	84	0.78
Luxembourg	0.13	0.16	0.46	0.036	2.5	1.6	0.7	64	73	30	88	6.14
Hungary	5.84	2.10	10.10	0.101	46.5	39.6	26.2	85	107	26	79	− 0.99
Malta	0.01	0.05	0.42	0.005	1.4	0.6	0.8	40	20	14	79	58.08
Netherlands	1.94	6.39	16.29	0.567	202.4	133.2	47.4	66	56	52	82	21.94
Austria	3.25	2.44	8.22	0.271	35.7	24.5	22.6	69	92	32	78	− 0.14
Poland	16.06	10.15	38.12	0.309	219.6	62.8	88.6	29	55	29	73	7.42
Portugal	3.80	2.02	10.54	0.163	73.8	19.1	25.9	26	31	28	80	13.25
Romania	14.10	4.93	21.80	0.124	61.5	40.3	60.4	66	97	22	76	− 0.31
Slovenia	0.50	0.51	2.00	0.035	8.6	4.9	6.4	57	77	38	73	− 2.82
Slovakia	1.94	0.74	5.41	0.055	9.9	13.8	8.3	139	129	28	86	− 0.40
Finland	2.28	1.16	5.25	0.180	32.8	10.7	13.1	33	54	25	77	7.43
Sweden	3.18	1.80	9.06	0.331	32.1	21.9	18.6	68	89	27	83	0.49
United Kingdom	17.29	14.27	60.44	2.049	207.3	114.3	122.7	55	75	22	80	4.21

**Table 2:** Correlation coefficients (*r*^2^) from linear regression analysis of factors potentially influencing P efficiency, P surplus and P losses in the food systems across the EU27 countries and their relationships to national data on mean soil Olsen-P and mean river-soluble reactive P (SRP) concentrations. System data are expressed on an areal basis. The results of regression analysis expressed on a per capita basis are given in Table S6. Asterisks give statistical significance: *P < 0.05; **P < 0.01; *** *P* < 0.001. *LU* livestock unit, *UAA* utilizable agricultural area, *GDP* gross domestic product. *NS* not significant (*P* > 0.05)

Dependent variable	Independent variable											
	Population density	Animal density	UAA	GDP	P imports				P surplus	P losses		
					System	Fertiliser	Feed	Food		System	Effluent	Soil
	(ca. ha^−1^)	(LU ha^−1^)	(ha)	(M€ ha^−1^)	(kg ha^−1^)				(kg ha^−1^)	(kg ha^−1^)		
P efficiency (%)												
System	NS	NS	NS	NS	NS	− 0.42***	NS	NS	− 0.25*	NS	NS	NS
Crop production	− 0.15*	− 0.16*	NS	NS	− 0.21*	− 0.66***	NS	NS	− 0.63***	− 0.16*	− 0.16*	NS
Animal production	0.43***	0.45***	NS	0.38**	0.52***	NS	0.62***	0.55***	NS	0.60***	NS	NS
Food processing	NS	NS	NS	NS	NS	NS	NS	NS	NS	NS	NS	NS
Surplus (kg P ha^−1^)	0.51***	0.54***	NS	0.30**	0.63***	0.79***	0.46***	0.54***	–	0.47***	0.36**	NS
Losses (kg P ha^−1^)												
System	0.83***	0.85***	NS	0.57***	0.89***	0.37**	0.86***	0.86***	0.47***	–	0.25*	NS
Effluent	0.28**	0.31**	NS	0.23*	0.34**	0.24*	0.19*	0.40***	0.18*	0.34**	–	NS
Soil	NS	NS	NS	NS	NS	NS	NS	NS	NS	NS	NS	–
Olsen-P (mg kg^−1^)												
Cropland	0.74***	0.85***	NS	0.79***	0.79***	0.36**	0.76***	0.75***	0.48**	0.76***	NS	0.22*
Grassland	0.49***	0.66***	NS	0.63***	0.63***	0.59***	0.47***	0.60***	0.64***	0.49***	NS	NS
River SRP (mg L^−1^)	NS	NS	NS	NS	NS	NS	NS	NS	NS	NS	0.31**	NS

Sector P efficiencies in crop production, livestock production and food processing ranged from 14 to 129 % (69 % overall), 14–52 % (24 % overall) and 62–88 % (78 % overall), respectively. The much lower P efficiencies associated with livestock production were because the large amounts of P recycled in manure were not classed as consumed or exported P outputs. The near tenfold variation in crop production P efficiency, which declined as areal fertiliser P imports increased (*P* < 0.001, *r*^2^ = 0.61), had a much larger influence on overall system P efficiency than either livestock P efficiency or food processing efficiency (Fig. 1D). Particularly low crop production efficiencies were recorded in Southern Europe because of larger areal P fertiliser inputs needed on the high proportion of calcareous soils in that region, and lower crop outputs due to the more limited water availability (Torrent et al. 2007). Countries with high animal production efficiencies had greater imports of animal feed and food which generated greater production output, and this was reflected in a significant positive relationship with GDP. Food processing efficiency was decreased as system P imports on a per capita basis increased (Table S6).

**Fig. 2:**
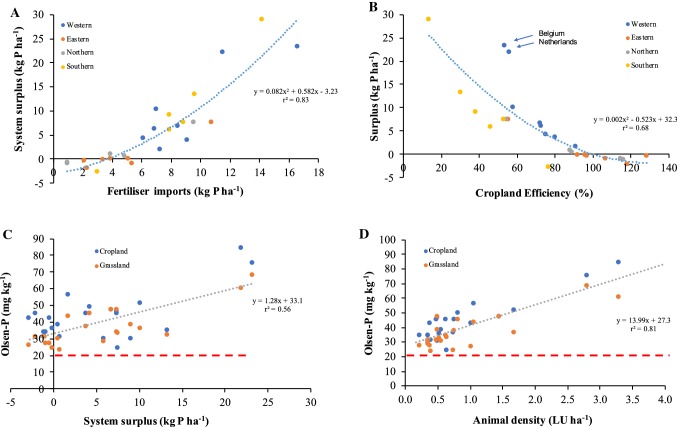
Fertiliser imports exerting a large influence on system surplus P across the EU 27 (**a**), which becomes zero only when cropland P efficiency across all Europe is 100 % (**b**). Belgium and the Netherlands have much higher P surpluses relative to their cropland efficiency because they have high animal densities. Countries with a high system P surplus accumulate more available P (measured as Olsen-P) in cropland and grassland soils, but this accumulation is at levels that are in excess of agronomic optimal requirements (red dotted line) (**c**). The large influence of animal density on system surplus is also reflected in their influence on soil Olsen-P concentrations (**d**). The Olsen-P data are from the EU LUCAS soil survey and represent mean values for soils in the EU 27 countries, excluding Malta (Tóth et al. 2013)

Excluding the small island of Malta, which had a disproportionally small land area (Table 1), the amounts of surplus P accumulating in the soil annually across Europe varied from − 3 to 29 kg P ha^−1^ year^−1^ or − 1.0 to 6.94 kg P ca^−1^ year^−1^ (representing up to 70 % of the P imported into the food system). These surpluses are in excellent agreement to the gross soil P balance for 2005 (− 7 to 28 kg P ha^−1^) estimated independently by the European Commission (Eurostat: https://ec.europa.eu/eurostat/web/products-datasets/-/t2020_rn310). Areal P surpluses were significantly positively correlated with system P inputs, especially fertiliser inputs (Fig. 2A), and declined as crop production efficiency and total system P efficiency increased (e.g. Fig. 2B). Animal density was also a significant (*P* < 0.001) cause of variation in system P surplus (Table 2). Largest P surpluses therefore occurred in Western Europe where P inputs and livestock densities were highest, especially in Belgium and the Netherlands (Fig. 2B), and this was again reflected in a significant positive relationship with GDP (Table 2). The results from the LUCAS soil survey showed that countries with higher P surpluses (and GDP) also tended to have greater Olsen-P concentrations in the soil (*P* < 0.001, Fig. 2C). Olsen-P concentrations ranged from 24 to 84 mg kg^−1^ (mean 43.3 mg kg^−1^, S.E. 3.03) in cropland soils and from 23 to 68 mg kg^−1^ (mean 36.3 mg kg^−1^, S.E. 2.44) in grassland soils. The link between soil Olsen-P and livestock density was particularly strong (Fig. 2D) due to the contribution from recycled manure P inputs.

**Fig. 3:**
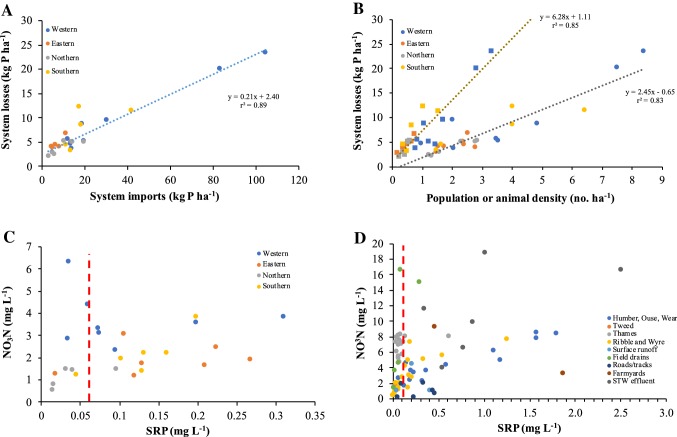
System P imports driving system total P losses (to water and to landfill) across the EU27 (**a**), with variation in P losses very largely explained by differences in animal and population densities that govern the P-input pressure (**b**). Mean soluble reactive P (SRP) and nitrate–N (NO_3_N) concentrations in rivers vary widely across Europe (**c**), and are largely in excess of P targets for eutrophication control (red dotted line). More extreme nutrient pollution of land runoff and individual rivers is typical of highly populated countries with intensive agriculture such as the UK (D). EU river data are from the Waterbase v14 nutrient survey (European Environment Agency 2018a, b) and data from the UK are from intensive catchment monitoring programmes (Withers et al. 2009; Neal et al. 2012)

Total P losses from the food system ranged from 2 to 23 kg P ha^−1^ and from 1.34 to 4.7 kg P ca^−1^, and increased markedly at a rate of 20 % of system P imports (Fig. 3A). Even with zero P imports, P losses averaged 2.60 kg ha^−1^. However, in contrast to system P surpluses, total P losses from the food system were governed more by feed and food imports (*P* < 0.001, *r*^2^ = 0.87) than by fertiliser imports, and were highly positively correlated equally to both population density and animal density (Fig. 3B). Together these two factors explained 93 % of the variation in system total P losses, which were consequently also strongly related to GDP. There was no relationship between Olsen-P levels and soil losses of P to water (Table 2). Total P losses to water were weakly related to both animal density and population density (*P* < 0.01, *r*^2^ = 0.3), but were not significantly related to the mean concentrations of SRP in rivers across the EU27 which ranged from 0.14 to 0.31 mg L^−1^ (Fig. 3C). River SRP levels were significantly higher where effluent losses were high (*P* = 0.003, *r*^2^ = 0.31). However, mean river SRP concentrations across Europe were generally in excess of the eutrophication control target, especially for individual rivers in regions receiving both high effluent P losses and land runoff from intensive farming (Fig. 3D; Table S3).

**Fig. 4:**
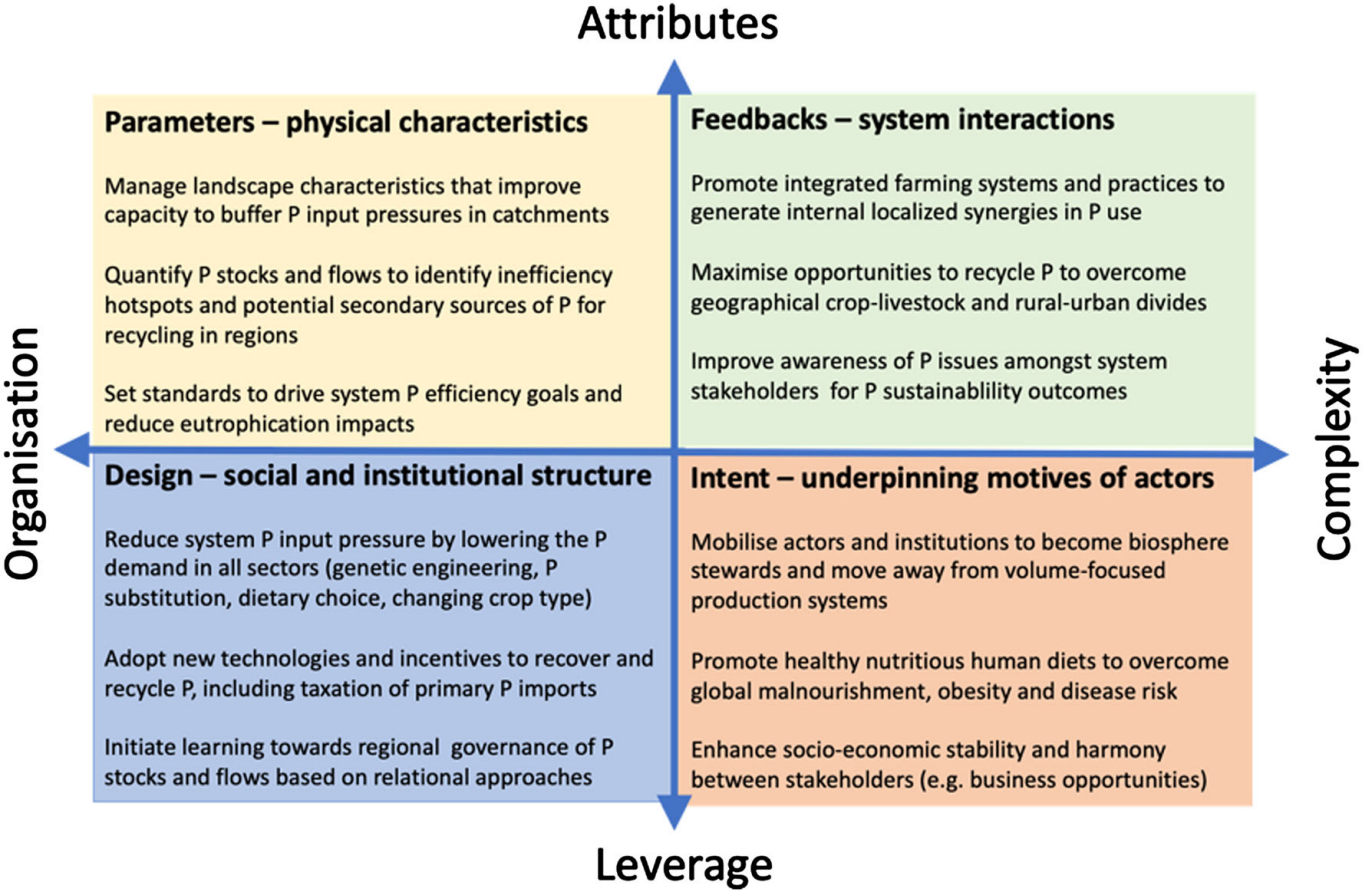
Towards an understanding of the social, economic and environmental dimensions of food systems (system attributes, organisation, complexity and leverage points) based on the management and governance of their parameters, feedbacks, design and intent. The framework provides selected examples of transdisciplinary management interventions to improve P-use efficiency and reduce eutrophication risk and is based on the conceptual thinking of Abson et al. (2016) and Gordon et al. (2017)

## Discussion

### Drivers of P inefficiency, surplus and loss

Substance flow analysis is a widely accepted and valuable model to compare stores and flows of P in complex systems after accounting for data uncertainty and their socioeconomic contexts (Chowdhury et al. 2014; Metson et al. 2015). The dataset produced by van Dijk et al. (2016) provided an opportunity to examine the P dynamics in food systems across Europe, and how they might relate to the dispersion and accumulation of P in their catchments, as assessed here by the LUCAS soil survey and the Waterbase river survey. Our analysis showed that high P-input pressure, either defined by area or per capita, reduced cropland and overall system P efficiency and increased system P surpluses and losses (Figs. 1, 2 and 3, Tables 2 and S6). Fertiliser P input was clearly the main driver of changes in system P efficiency through its effect on cropland efficiency. A reduction in average fertiliser use of 1 kg ha^−1^ across Europe gave a 6 % increase in cropland P efficiency and a 5 % increase in system P efficiency in 2005. Multiple regression analysis indicated that 95 % of the variation in system P imports was explained solely by differences in animal density to meet the population demand for meat and dairy products. In addition to animal feed imports, high fertiliser P imports are needed to meet this demand because, across Europe as a whole, 63 % of crop production P output is fed to livestock. In many EU countries, fertiliser P inputs are therefore still too high relative to the productive P output, and this oversupply is also reflected at a global scale (Helin and Weikard 2019).

The largest surpluses and losses of P were in Western Europe with both high population density and intensive animal agriculture and this was reflected in strong links to national wealth (GDP). Surpluses were most strongly linked to fertiliser inputs as the dominant P import, and above an apparent minimum average fertiliser P demand across Europe of 4 kg P ha^−1^, the regional P surplus increased by 1.9 kg ha^−1^ for every additional kg of fertiliser used (Fig. 2A). A strong link between fertiliser P inputs and P surplus at national scale has also been shown in temporal analyses of national P budgets (Withers et al. 2014, 2018). Those countries with the largest P imports, and animal densities that determine areal manure P loadings to agricultural land, also had the highest concentrations of Olsen-P in both cropland and grassland soils because of the surpluses they generated (Figs. 2, 3). This link to soil P status was significant even though Olsen-P is an indicator of cumulative surplus P inputs over many years rather than those generated in only 1 year.

Total losses of P from the food system were high (ca. 20 % of inputs) and reflect the multiple hotspots of P cycling within the food chain that increase P loss vulnerability: wastewater treatment, slaughter and food waste, manure handling and land management. The lack of a significant relationship between P losses to water and mean river SRP concentrations across Europe is perhaps not surprising given the complex seasonal transfers and cycling of P across the land–water interface. Highly variable spatial and temporal patterns of P delivery and retention of different particulate and dissolved forms of P during storm events and complex lag patterns of SRP release from legacy P stores in the landscape make it very difficult to identify single causal factors (Powers et al. 2016; Dupas et al. 2018; Withers and Bowes 2018). The Waterbase survey did not have sufficient data on total P concentrations to allow cross-country comparisons to food system P flows. The significant but weak relationship between effluent losses (areal or per capita basis, Tables 2 and S6) and mean river SRP levels is however consistent with previous work that has demonstrated the beneficial impact of lowering effluent losses on river SRP concentrations across Europe (e.g. Foy 2007; European Environment Agency 2015). The wide dispersal of the anthropogenic P imported and circulating in the food system is exemplified by the highly variable, elevated nutrient signals found in both soils and waters, and this will likely become exaggerated by climate change (Forber et al. 2018).

### An agronomic issue or a wider food chain issue?

Overuse of P inputs relative to food P demand is not only wasteful of critical phosphate rock resources but also generates surpluses and losses of unused P that are damaging our environment. Europe is almost entirely dependent on imports of P to secure its food supply, yet its overall P-use efficiency in 2005 was low (38 %) and the average P surplus and P loss from the EU food system was ca. 5 and 6 kg P ha^−1^, respectively. Food system P inefficiencies occur across multiple scales ranging from agronomic inefficiencies at field and farm scale to wider societal inefficiencies associated with the regional organisation of food systems, and the complex long food supply chains that have emerged as a consequence of consumer demand and global trade (Box 1). Improving the efficiency and sustainability of P use and reducing eutrophication impacts relies on overcoming both agronomic and wider food supply chain P inefficiencies to minimise P surpluses and losses. However, only agronomic P inefficiency is currently addressed to any degree in P source management strategies, and there is a fundamental governance disconnect between the management of P on individual farms and the management of P at regional or national scale (Leinweber et al. 2018).

**Box 1:** Inefficiencies of phosphorus use in food systems operate at multiple scales

**Agronomic P inefficiencies**	
Inherent inefficiencies in nutrient use arise at field and farm scale because of (a) the natural immobilisation of P by biotic (i.e. microbial) and abiotic (i.e. physicochemical) processes in soils that compete with plants for the nutrients available, (b) uncertainties in the prediction of economically optimal amounts of P required by different crops and animals in different seasons, and (c) leakage of P from soils along different hydrological pathways into surface and groundwaters. Agronomic inefficiencies are influenced by farmer decisions on land use, land management and nutrient inputs and the landscape characteristics that determine P mobility. Large P inefficiencies will arise on P-fixing soils, when P inputs deviate from crop and animal P demand or output, and due to a mismatching of production practices with land capability and runoff risk fuelled by food production subsidies. Rural landscapes have been abused by modern farming practices that have degraded the ecosystem attributes that determine the future resilience of food production systems to global stressors (e.g. climate, market and resource shocks).	
**Wider food chain P inefficiencies**	
Inherent inefficiencies in nutrient utilisation also occur at other stages in the food supply chain because the recirculation of nutrients between crops, animals and humans has become disrupted over space and time in unintended ways by (a) the specialisation and industrialisation of agriculture that has geographically segregated crop and animal production systems, (b) by the extensive urbanisation and international trade that has preferentially concentrated nutrients into urban areas with little of those nutrients returned to where the food is produced, (c) general economic growth and affluence that has favoured meat-rich diets and (d) wastage related to pre-farm gate P supply chains including the P losses that occur during mining, fertiliser processing, storage and transport (depending on the country of production and length of the supply chain). Such broad infrastructure changes have resulted in considerable imbalance in nutrient flows between and across regions, and shifts in the types of food being consumed. These society-driven P inefficiencies have occurred due to the highly variable economic and social development of agriculture and related markets in different regions, and have been evolving for some considerable time. These wider P inefficiencies are more important in determining eutrophication risk than agronomic inefficiencies, because they are largely responsible for the chaotic accumulation of P within the landscape.	

In recent decades, a combination of voluntary and regulatory guidelines, measures and decision support tools have been introduced to encourage best management practices to improve agronomic P efficiency, and reduce land runoff and soil erosion risk (e.g. Kleinman et al. 2015). This places the burden of responsible P management on the farmer and landowner, and is often undermined by farmers’ innate aversion to risking practices that might lower yields, which means that they have a propensity to apply excess P to offset uncertainty (Buckley and Carney 2013). A further underlying issue confounding sole reliance on the success of agronomic solutions is that their site-specific nature makes them variably cost-effective and liable to failure during extreme events (e.g. Ockenden et al. 2017).

In contrast to agronomic inefficiency, the wider supply chain P inefficiencies associated with food production, processing, retailing and consumption are not wholly addressed, although strategic frameworks to foster sustainable P use across multiple scales and stakeholder groups have been proposed (Cordell and Neset 2014; Metson et al. 2015; Withers et al. 2015). From a complex systems perspective, a whole system cannot be sustainable if only a sub-system is optimised. Food systems are generating P surpluses at catchment, regional and national scales because the home-grown and imported nutrients consumed and excreted by animals and humans are not uniformly balanced with cropland demand at a regional or national scale (Box 1). This is not a new phenomenon as regional nutrient imbalances have been in existence for at least two centuries (e.g. DeGraef 2017), but the scale of the problem has become more acute with urbanisation and the globalisation of feed and food supply (MacDonald et al. 2011; Gordon et al. 2017). Current policies try to address P imbalance only at an individual farm scale. Our analysis suggests that achieving a zero P surplus at regional scale would lead to a more P efficient food system, and reduce total P losses by ca. 35 %. The relationships between system P inputs and P surplus indicate an overall reduction in system P imports of ca. 40 % from the 2005 level, largely as fertiliser, is necessary to deliver zero P surplus across Europe.

Recent initiatives towards more sustainable agricultural development via a circular P economy will help to close the P cycle and reduce dependence on P imports (Jurgilevich et al. 2016; Withers et al. 2018). However, the potential for P recovery and recycling is currently being left to the open market, and maybe confounded by variable economic, agronomic performance and/or regulatory restrictions. Consequently, regional P imbalances persist. Similarly, there is no apparent policy awareness of the need to reduce P demand pressure across the whole food system to improve overall P efficiency and reduce surplus P accumulation and subsequent losses (Fig. 2A). Europe has reduced its consumption of imported P fertilisers by ca. 15 % since 2005, and this will have increased system efficiency by 5 %, reduce system P surplus by 35 % and reduce total P loss by 5 % (Figs. 1C, 2A, and 3A). This reduction in P use was in response to a market shock (the price of phosphate rock rose over 800 % in 2008), rather than a concerted drive towards greater P-use efficiency, and lower P inputs cannot be sustained without also reducing P demand if long-term food productivity is not to be compromised (Withers et al. 2018). Lowering P demand might be best achieved by reducing livestock densities since this appears as the main driver of total system P imports (Fig. 1A). Our analysis suggests a 20 % reduction in livestock density across Europe would stimulate lower system P imports by ca. 3 kg ha^−1^, which in turn would help to reduce total system P losses by at least 0.6 kg ha^−1^, (Figs. 1A, 3A).

Collective actions across all sectors of the food chain are therefore needed to reduce and manage both P supply and demand pressures in order to resolve the regional P imbalances causing environmental damage (Cordell and White 2014; Springmann et al. 2018). Such collective action requires transdisciplinary approaches to flexible decision-making, embracing a diversity of ‘knowledge systems’ and values, an analysis of leverage interfaces within and between sub-systems of the food chain, and of interactions between stakeholders, and an understanding of their adaptive and transformative capacities (Reed 2008; Jacobs et al. 2017; Ruben et al. 2019).

### Developing P-sustainable systems

Following the conceptual framework of Abson et al. (2016), we can consider two basic transdisciplinary approaches to manage P more sustainably across the whole food system. One approach is to better manage the parameters and feedbacks of the existing food system, whilst another approach is to reorientate the food system through more transformative adaptations in system design and intent (Fig. 4). System parameters and feedbacks include the P stores and flows in the food system, operating internal synergies towards P recycling, and social response to policy measures such as the introduction of agri-environment schemes (Abson et al. 2016). System adaptations to manage parameters and feedbacks might include: reducing the unnecessary use of imported P, for example, by omitting P fertiliser where soil Olsen-P exceeds the agronomic optimal value (Fig. 2C); maximising opportunities to recycle existing bioresources as fertiliser substitutes, or the introduction of P efficiency standards to drive more sustainable P use (Fig. 4). Appropriate geographical scales for P governance of parameters and feedbacks are catchments and regions, where: (a) the multifunctionality of landscapes can be managed to minimise chronic P losses and optimise the balance of ecosystem service provision, (b) P stores and flows can be quantified to identify hotspots of societal P inefficiency and (c) business opportunities to recover and recycle P can be identified to overcome arable-livestock and rural–urban P imbalances (Doody et al. 2016; MacDonald et al. 2016; Powers et al. 2019). Improving P efficiency and achieving zero P surplus at the regional scale requires assessment of minimum regional P demand based on food production needs (e.g. Helin and Weikard 2019), taking full account of legacy P stores in the soil, and of regional accessibility to secondary P resources that can substitute for P fertiliser and feed imports.

According to Abson et al. (2016), management interventions relating to the parameters and feedbacks in a system may have too shallow leverage to lead to sufficient beneficial change: for example, they do not alter the organisation and total P demand of the food system, and therefore the P-input pressure that governs overall environmental vulnerability remains (Figs. 2A, 3A). The continuing poor state of ecosystems despite considerable policy efforts to improve them suggest more transformative change is required (e.g. Díaz et al. 2019). For example, pollution is still a major cause of the failure of over 60 % of surface waters in Europe to achieve good ecological status (European Environment Agency 2018a). Transformative change requires a radical reassessment of system design and intent to lower P demand, which stakeholders are best placed to influence and enact the necessary leverage and how best to achieve the transformation. For example, Termeer et al. (2017) suggests that system transformation is best achieved through deep continuous change rather than wide-scale unplanned system disruption. Changes in system design might include growing the type of crops and foods that are best aligned with healthy diets, including lowering meat intake, new technologies to allow alternatives to the P additives used during food processing, or facilitating regional governance through mutual learning based on evidence gathering and experience of what works and what does not (Fig. 4). This includes ensuring system actors recognise their ‘connectedness’ to other system actors and components (McNamee and Gergen 1999).

Reorientation of system intent requires a re-examination of stakeholder motives values, power and influence, and the potential to move away from the current resource hungry economic model that values food volume more than food quality, or its sustainable production, at the expense of the environment (Jurgilevich et al. 2016; Gordon et al. 2017). Changes in intent might be enacted by reconnecting people with nature, and encouraging High Nature Value (HNV) farmers who operate on lower inputs to provide a wider range of ecosystem services benefits, such as biodiversity, in addition to their core farming activities (Lomba et al. 2014). Large stakeholders who have influenced the organisation of the food system, such as supermarkets with their own brands and supply chains (Burch and Lawrence 2005), have the potential to lever consumer preferences and production patterns in support of healthier diets and environmental integrity. For example, reducing regional P imbalance by sourcing locally produced food using secondary P inputs (Cordell and Neset 2014).

Research is needed to characterise the social capital and transition pathways towards such transformative change in different regions and environmental settings, taking account of synergies and trade-offs with other cycles (e.g. C, N and Water) (e.g. Metson et al. 2015; Jacobs et al. 2017). Transition pathways to address regional P imbalance must therefore disentangle the role of all stakeholders (not just farmers and landowners) on the basis of their transformational capacity and empower them to take responsibility in the collective governance of P beyond the farm gate. It could even be argued that truly sustainable food systems should also consider the environmental provenance of food and feed P imports beyond regional geographical boundaries (Lathuillière et al. 2014). Avoiding the traps of reductionist, agronomic-centric solutions to P inefficiency and pollution risk therefore means creating fair and transformative polycentric governing strategies based on a deep understanding of P dynamics at global to regional scales on the one hand, and stakeholder roles, interests, and capacities on the other. In addition to topdown policy makers, key stakeholders to enlist in transition strategy formation include farmers, local and national environmental managers, water companies, agri-businesses, charities, and other organisations with diverse but important system roles (Morrison et al. 2019).

## Conclusion

Phosphorus is one of the biogeochemical flows from our food systems that is causing widespread environmental damage and concerns over future food security due to P scarcity. Inefficiencies in P use across time and space, surplus P accumulation in rural and urban environments and current production and waste disposal practices are all contributing to accelerated P losses and eutrophication of waterbodies and reduced resilience of the food system to environmental, market or resource shocks. A comparison of stores and flows of P across Europe have shown that P-input pressures linked to population pressure and demand for meat and dairy products are driving highly variable P inefficiencies across multiple scales, surplus P accumulation in catchments and large losses to water and landfill. In particular, fertiliser P inputs are still too high in relation to productive P output and appear as the dominant driver of system P surpluses across Europe. These P inefficiencies and imbalances occurring across the whole food system are not currently being adequately managed because improving P-use efficiency and reducing system P losses is seen as an agronomic issue rather than a wider food chain issue. Resolving the disorderly disruptions to the P cycle (P chaos) created by our food systems and improving their environmental performance therefore requires a shift in research agendas to focus on the whole food system and its P demand, and not just on landscape P delivery to adjacent waterbodies, or placing the burden of responsibility solely on producers. The environmental performance of food systems can only be improved by tackling the wider food chain inefficiencies that reflect societal functioning, but this requires a societal response and stakeholder interaction in addition to the current agronomic solutions. A reorientation of design and intent of the wider food system, and better management of system parameters and feedbacks alongside transdisciplinary polycentric governance of P, is needed to lower P demand and deliver more P efficient and P-sustainable food production from local to global scales. The interdependencies of scale covering both biophysical and socioeconomic aspects of system P use need to be considered in more detail for this reorientation to occur.

## Electronic supplementary material

Below is the link to the electronic supplementary material.

Supplementary material 1 (PDF 312 kb)
