# Supplementary material for: Towards resolving the phosphorus chaos created by food systems
Source: Ambio. 2019 Sep 21;49(5):1076–89. doi: 10.1007/s13280-019-01255-1 (PMC7067724; doi:10.1007/s13280-019-01255-1)
Supplement: Supplementary file 1 — Supplementary material 1 (PDF 312 kb) [file 13280_2019_1255_MOESM1_ESM.pdf]

Ambio

Electronic Supplementary Material
This supplementary material has not been peer reviewed

Title: **Towards resolving the phosphorus chaos created by food systems**

Paul J.A. Withers, Kirsty G. Forber, Christopher Lyon, Shane Rothwell, Donnacha G. Doody, Helen P. Jarvie, Julia Martin-Ortega, Brent Jacobs, Dana Cordell, Myles Patton, Miller A. Camargo-Valero, Rachel Cassidy

Table S1. Allocation of EU countries to four regions according to their climate zones (Commission of the European Communities 2008).

Western Europe	Belgium, Denmark, France, Germany, Ireland, Luxembourg, Netherlands, United KIngdom
Eastern Europe	Austria, Bulgaria, Czech Republic, Hungary, Poland, Romania, Slovakia
Northern Europe	Estonia, Finland, Latvia, Lithuania, Sweden
Southern Europe	Cyprus, Greece, Italy, Malta, Portugal, Slovenia, Spain,

Table S2. Descriptive statistics for national Olsen-P concentration (mg kg^{-1}) data in cropland and grassland across Europe. Data are from the LUCAS survey (Toth et al. 2013).

**Country**	**Crop type**	**Minimum**	**Maximum**	**Mean (s.e.)**	**Median**	**n**
Belgium	Cropland	18	139	74.5 (5.21)	78.5	33
	Grassland	26	153	68.4 (10.03)	60.6	14
Czech Republic	Cropland	11	432	44.8 (2.23)	40.4	224
	Grassland	10	88	30.6 (2.07)	27.7	67
Denmark	Cropland	13	154	51.3 (1.92)	46.8	166
	Grassland	15	79	35.7 (3.89)	31.8	20
Germany	Cropland	10	212	55.9 (0.95)	52.4	934
	Grassland	10	226	42.7 (1.49)	33.6	427
Estonia	Cropland	10	97	34.1 (2.62)	29.4	54
	Grassland	11	85	30.8 (4.50)	21.9	26
Ireland	Cropland	10	144	46.6 (11.91)	41.8	10
	Grassland	10	200	46.9 (3.11)	31.4	149
Greece	Cropland	10	116	30.7 (1.93)	23.9	119
	Grassland	10	49	28.2 (3.82)	25.4	12
Spain	Cropland	10	218	30.2 (0.72)	22.1	1069
	Grassland	10	342	38.0 (3.61)	28.1	121
France	Cropland	10	215	45.5 (0.70)	40.1	1497
	Grassland	10	225	37.2 (1.11)	28.6	611
Italy	Cropland	10	294	36.7 (1.92)	24.0	385
	Grassland	10	170	33.1 (3.47)	19.9	83
Latvia	Cropland	11	109	34.0 (2.51)	28.6	73
	Grassland	11	115	27.8 (2.37)	23.0	62
Lithuania	Cropland	10	185	31.0 (2.20)	24.5	121
	Grassland	10	95	23.4 (1.70)	18.9	81
Hungary	Cropland	10	276	34.0 (1.78)	26.2	257
	Grassland	12	181	31.5 (6.59)	21.8	26
Netherlands	Cropland	30	149	83.7 (2.79)	82.7	100
	Grassland	11	139	60.4 (3.59)	56.7	73
Austria	Cropland	10	136	36.0 (1.93)	29.2	144
	Grassland	10	128	24.4 (1.73)	19.3	109
Poland	Cropland	10	205	44.7 (0.88)	40.2	815
	Grassland	10	288	34.5 (1.87)	26.3	232
Portugal	Cropland	10	120	34.9 (4.14)	22.9	42
	Grassland	10	129	31.5 (7.06)	20.4	18
Slovenia	Cropland	14	110	42.4 (11.94)	35.6	7
	Grassland	11	91	26.3 (4.65)	22.1	17
Slovakia	Cropland	10	185	42.1 (2.69)	35.1	112
	Grassland	11	73	26.6 (2.96)	22.4	30
Finland	Cropland	10	186	46.0 (1.38)	42.9	302
	Grassland	12	103	46.9 (5.20)	40.8	19
Sweden	Cropland	10	169	38.2 (2.06)	27.8	175
	Grassland	10	132	30.5 (2.45)	23.2	86
United Kingdom	Cropland	10	403	49.3 (2.03)	41.0	329
	Grassland	10	284	45.2 (1.94)	33.9	356

Table S3. Descriptive statistics for national river soluble reactive P concentrations data ($\mu g\ L^{-1}$) reported to the European Commission as part of the Waterbase v14 survey (European Environment Agency 2018).

Country	Minimum	Maximum	Mean (s.e.)	Median	Stations[1]	Samples[2]
					n	n
Belgium	8	3811	310 (28.1)	194	47	3199
Bulgaria	5	2402	209 (13.1)	118	86	3833
Czech Republic	10	657	105 (4.9)	76	71	4433
Denmark	9	465	59 (3.8)	51	42	5305
Germany	3	369	72 (2.2)	57	142	12109
Estonia	2	249	31 (2.0)	24	44	2127
Ireland	3	151	34 (2.3)	30	29	896
Greece	10	4890	310 (48.5)	150	28	794
Spain	<1	2940	160 (11.0)	33	200	10688
France	2	4550	74 (2.9)	37	671	34867
Italy	2	5140	131 (6.9)	48	323	16371
Cyprus[2]	4	3727	102 (50.4)	7	17	563
Latvia	4	272	40 (2.5)	25	51	2445
Lithuania	11	2148	96 (19.4)	45	31	1834
Luxembourg	16	59	35 (3.3)	36	3	148
Hungary	5	2396	223 (15.3)	84	97	10665
Netherlands	35	216	94 (5.9)	78	15	999
Austria	<1	214	18 (0.91)	8	154	8897
Poland	8	4517	267 (19.7)	136	130	7438
Portugal	11	2251	128 (21.1)	50	32	1636
Romania	1	1831	119 (8.9)	51	110	5088
Slovenia	1	812	44 (8.8)	14	25	915
Slovakia	3	1693	128 (12.2)	70	44	2438
Finland	<1	108	15 (0.8)	5	148	6352
Sweden	1	258	14 (0.9)	6	115	7000
United Kingdom	2	3548	198 (9.8)	50	280	17003

[1]Number of river stations sampled annually for the reporting period of analysis (2003-2007 or nearest to that period).
[2]Total number of samples over the whole reporting period represented by the analysis.

Table S4. Descriptive statistics for soluble reactive P concentration (µg L^{-1}) monitored in different rivers in lowland England. Data are from Neal et al. (2012).

River[1]	Sampling Station	Minimum	Maximum	Mean (s.e.)	Median	n
Aire	Beal Bridge	72	2579	1107 (49.8)	1165	159
Calder	Methley Bridge	37	2699	1176 (55.0)	1250	145
Derwent	Bubwith	26	370	146 (7.1)	137	136
Don	Sprotborough	38	3505	1578 (72.5)	1510	140
Great Ouse	Great Paxton	222	3171	1580 (106.5)	1534	58
Nidd	Skip Bridge	38	1687	591 (30.9)	587	143
Ouse	Acaster Malbis	32	1150	368 (25.6)	271	143
Ouse	Clifton Bridge	37	942	227 (13.5)	188	145
Swale	Catterick Bridge	16	1050	127 (11.2)	95	131
Swale	Thornton Manor	29	578	205 (10.3)	183	140
Trent	Cromwell Lock	235	3230	179 (72.1)	197	112
Tweed	Boleside	1	272	46 (5.6)	26	106
Tweed	Norham	1	366	33 (3.5)	28	108
Tweed	Ormiston Mill	<1	168	37 (2.6)	29	106
Ure	Boroughbridge	2	170	62 (3.3)	53	138
Wear	Sunderland Bridge	65	666	322 (26.7)	287	55
Wharfe	Tadcaster	23	466	192 (10.2)	161	149
Thames	Wallingford	117	210	618 (28.0)	480	256
Lambourn	Boxford	46	225	116 (5.1)	110	76
Lambourn	East Shefford	10	78	34 (1.3)	29	81
Lambourn	Shaw	55	228	96 (2.4)	95	81
Pang	Bucklebury	39	1005	140 (14.3)	108	83
Pang	Blue Pool	16	95	45 (2.2)	39	83
Pang	Frilsham	20	313	63 (4.5)	59	83
Pang	Tidmarsh	7	410	65 (4.5)	55	137
Dun	Hungerford	19	122	49 (3.1)	44	33
Kennet	Clatford	42	444	89 (11.3)	65	52
Kennet	Fobney	33	128	69 (3.4)	70	33
Kennet	Hungerford	27	150	51 (3.5)	47	33
Kennet	Mildenhall	40	282	71 (5.8)	58	51
Kennet	Woolhampton	27	258	103 (4.6)	93	121
Kennet/Avon	Hungerford	8	218	79 (8.5)	81	35
Pendle Water	Barrowford	1	50	22 (1.7)	23	43
Colne Water	Barrowford	19	1950	172 (46.8)	88	43
Calder	Aldam Bridge	17	1020	139 (25.4)	89	43
Hyndburn	Hyndburn Brook	4	122	46 (4.4)	43	43
Calder	Whalley	53	751	194 (23.5)	162	43
Yarrow	Chorley	1	73	36 (2.9)	38	43
Douglas	Adlington	78	801	206 (23.7)	156	43
Douglas	Standish	72	813	182 (21.8)	141	44
Douglas	Parbold	4	109	37 (3.9)	32	43
Tawd	Hoscar	23	144	73 (4.6)	70	43
Eller Brook	Briars Lane	110	964	340 (34.5)	245	43
Douglas	Waynes Blade Bridge	212	2600	1251 (90.4)	1170	43
Ribble	Dunsop	1	12	5 (3.6)	5	41
Hodder	Lower Hodder	2	37	19 (1.4)	20	44
Loud	Mytham Bridge	23	495	68 (11.5)	53	41
Ribble	Gisburn	16	259	61 (6.4)	49	44
Ribble	Great Mitton	12	120	45 (3.1)	43	42
Ribble	Ribchester	41	607	111 (14.9)	77	44
Darwen	Roach Bridge	152	1530	547 (48.8)	488	44
Wyre	Marshaw Bridge	1	18	6 (0.5)	7	42

Wyre	Stoops Bridge	1	29	7 (0.8)	6	40
Wyre	Abbeystead Weir	2	22	8 (0.7)	7	42
Wyre	Garstang Bridge	4	38	20 (14.8)	21	44
Calder	Calder Bridge	3	147	28 (4.4)	22	44
Brock	Bilsborough	3	116	44 (4.1)	37	43
Wyre	St Michaels	48	571	163 (17.0)	130	44

[1]Rivers were allocated to river basins as follows:
Humber, Ouse and Wear basins included Aire, Calder, Derwent, Don, Great Ouse, Nidd, Ouse, Swale, Trent, Ure, Wear and Wharfe rivers
Tweed basin included just the Tweed river.
Thames basin included Dun, Kennet, Kennet/Avon, Lambourn, Pang and Thames rivers.
Ribble and Wyre basins included Brock, Calder, Colne Water, Darwen, Douglas, Eller Brook, Hodder, Hyndburn, Loud, Pendle Water, Ribble, Tawd, Wyre and Yarrow rivers.

Table S5. Descriptive statistics for soluble reactive P concentration (µg L^{-1}) monitored in different types of land runoff and effluent discharge in lowland England. Data are from Withers et al. (2009) and Neal et al. (2012).

**Runoff type**	**Catchment site**	**Minimum**	**Maximum**	**Mean (s.e.)**	**Median**	**n**
Surface runoff	Avon	70	1645	405 (250.5)	161	6
	Dinedor	7	412	207 (30.2)	217	13
	Loddington	8	92	43 (6.1)	41	16
Field drains	Avon	4	39	(22 (7.7)	22	4
	Kivernoll 1	7	251	82 (12.7)	67	22
	Kivernoll 2	5	487	294 (51.9)	342	10
	Dinedor	4	51	17 (5.9)	14	7
	Loddington	7	344	65 (24.4)	32	13
Roads	Avon	57	618	230 (43.2)	185	14
	Kivernoll	56	1088	335 (80.1)	156	17
	Whitchurch	4	1330	445 (128.5)	425	12
	Welland	15	266	58 (29.9)	30	8
Farm tracks	Avon	84	925	460 (155.2)	522	5
	Loddington	22	270	94 (24.5)	46	10
Farmyards	Rosemaund	61	5680	1872 (556)	1380	11
	Kivernoll	75	1179	462 (88.8)	489	13
Septic tank effluent	Dinedor	311	1388	774 (91.7)	772	13
	Loddington	123	1230	542 (346.6)	274	3
	Rosemaund	1086	3320	2508 (713.7)	3120	3
UWWTC effluent[1]	Hungerford	13	4170	1010 (144.7)	585	51
	Marlborough	179	1104	341 (26.7)	287	53
	Newbury	401	1970	878 (50.9)	813	53

[1]Urban Wastewater Treatment Centre

Table S6. Correlation coefficients (r^2) from linear regression analysis of factors potentially influencing P efficiency, P surplus and P losses in the food system across the EU27 countries and their relationship to national data on mean soil Olsen-P and mean river soluble reactive P (SRP) concentrations. System data are expressed on a per capita basis. The results of regression analysis expressed on an areal basis are given in Table 2 in the main text. Asterisks give statistical significance: * P <0.05; **P <0.01; *** P <0.001.

Dependent variable	Independent variable											
	Population density	Animal density	UAA[2]	GDP[3]	P imports				P surplus	P losses		
					System	Fertiliser	Feed	Food		System	Effluent	Soil
	(ca. ha^{-1})	(LU[1] ca^{-1})	(ha ca^{-1})	(M€ ca^{-1})	(kg ca^{-1})				(kg ca^{-1})	(kg ca^{-1})		
P efficiency (%)												
System	NS	NS	NS	NS	-0.28**	-0.39**	NS	NS	-0.62***	-0.16*	NS	NS
Crop production	- 0.15*	NS	NS	NS	-0.23*	NS	NS	-0.23*	-0.54***	NS	NS	NS
Animal production	0.43***	NS	NS	NS	NS	NS	0.24*	0.39**	NS	NS	NS	NS
Food processing	NS	0.38**	NS	NS	-0.34**	-0.5***	0.17*	NS	-0.46***	-0.60***	NS	-0.37**
Surplus (kg P ca^{-1})	NS	0.37**	NS	NS	0.64***	0.54***	0.23*	0.21*	-	0.26**	NS	NS
Losses (kg P ca^{-1})												
System	NS	0.65***	NS	NS	0.59***	0.27**	0.56***	NS	0.26**	-	NS	0.50***
Effluent	NS	NS	NS	NS	NS	NS	NS	NS	NS	NS	-	NS
Soil	NS	0.40***	0.17*	NS	NS	NS	NS	NS	NS	0.50***	NS	-
Olsen-P (mg kg^{-1})												
Cropland	0.74***	NS	-0.20*	0.22*	0.26*	NS	0.30**	0.57***	NS	NS	NS	NS
Grassland	0.49***	NS	NS	0.31*	0.35**	NS	NS	0.53***	0.18*	NS	0.25*	NS
River SRP (mg L^{-1})	NS	NS	NS	NS	NS	NS	NS	NS	NS	NS	0.27**	NS

[1]LU: Livestock unit; [2]UAA: Utilizable agricultural area; [3]GDP: Gross domestic product; NS: Not significant (P >0.05).

Figure S1. Schematic of the main P stores and flows in Europe's food system and definitions of the metrics used to assess food system efficiency, surplus accumulation in soil and system P losses. Productive output is coloured green, losses are coloured red.

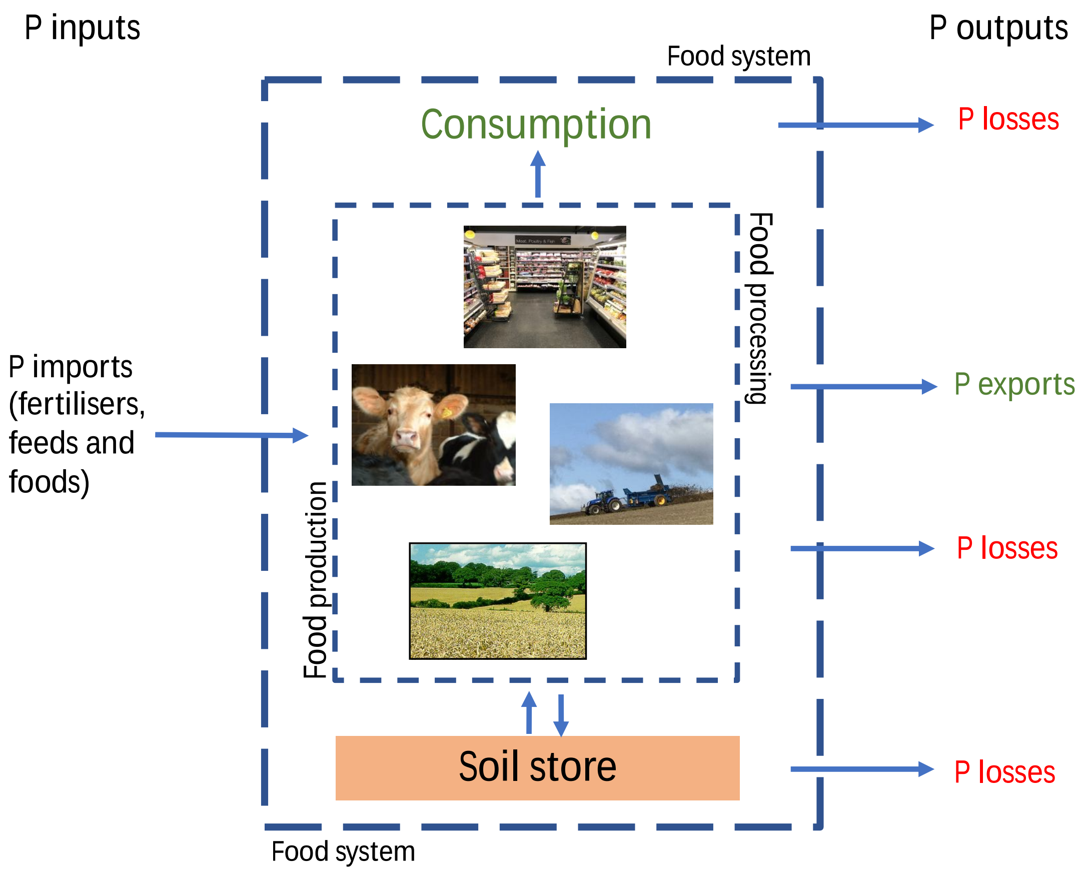

Metric definitions

System efficiency = (human P consumption + P exports) / P imports

System surplus = total P inputs – total P outputs (P exports + P losses)

System losses = human consumption + food production and processing + soil

References

Commission of the European Communities. 2008. Regions 2020: An Assessment of Future Challenges for EU Regions. Commission Staff Working Document. Available at: https://ec.europa.eu/regional_policy/en/information/publications/studies/2008/regions-2020-an-assessment-of-future-challenges-for-eu-regions

European Commission. 2007. *Eurostat Pocketbooks: Agriculture Main Statistics 2005-2006*. Luxembourg:Office for Official Publications of the European Communities.

European Environment Agency. 2018. Nutrients in Freshwaters in Europe. Available at: https://www.eea.europa.eu/data-and-maps/indicators/nutrients-in-freshwater/nutrients-in-freshwater-assessment-published-6

Neal, C., M. Bowes, H.P. Jarvie, P. Scholefield, G. Leeks, M. Neal, P. Rowland, H. Wickham, et al. 2012. Lowland river water quality: A new UK data resource for process and environmental management analysis. *Hydrological Processes* 26(6): 949-960.

Tóth, G., A. Jones, and L. Montanarella, (Eds.). 2013. *LUCAS Topsoil Survey. Methodology, Data and Results.* JRC Technical Reports. Publications Office of the European Union, EUR26102 – Scientific and Technical Research Series, Luxembourg.

Withers, P.J.A., H.P. Jarvie, R.A. Hodgkinson, E.J. Palmer-Felgate, A. Bates, M. Neal, R. Howells, C.M. Withers, and H. Whickham. 2009. Characterization of phosphorus sources in rural watersheds *Journal of Environmental Quality* 38: 1998-2011.
